# The context of violent disagreements between parents: a qualitative analysis from parents’ reports

**DOI:** 10.1186/1471-2458-14-1324

**Published:** 2014-12-24

**Authors:** Megan H Bair-Merritt, Mahua Mandal, Norman B Epstein, Carol A Werlinich, Deanna Kerrigan

**Affiliations:** Division of General Pediatrics, Boston Medical Center, 88 East Newton Street, Vose 305, Boston, MA 02118 USA; Carolina Population Center, University of North Carolina, Chapel Hill, NC USA; Department of Family Science, School of Public Health, University of Maryland, College Park, MD USA; Department of Health, Behavior and Society, Johns Hopkins Bloomberg School of Public Health, Baltimore, MD USA

**Keywords:** Intimate partner violence, Qualitative study, Children exposed to intimate partner violence, Household labor, Parenting, Substance use

## Abstract

**Background:**

Intimate partner violence (IPV) is a prevalent public health problem that affects millions of families. Much of what is known about IPV comes from quantitative studies that often "count" acts of IPV without exploring in depth the circumstances surrounding the violence, thereby leaving critical questions unanswered; existing qualitative studies tend to focus solely on women’s perspectives. There is a dearth of dyadic qualitative research exploring the context of IPV in families with children, thus hindering the development of effective interventions for families experiencing IPV.

**Methods:**

Seven heterosexual couples were recruited from a University-based family therapy clinic to participate in qualitative interviews. Couples were eligible if they had experienced severe verbal or any physical aggression during the past 4 months; had ≥ one child living in the household; were English-speaking; and were ≥ 18. Each individual was interviewed separately. Key topics explored included specific types of violence used by men and women; primary triggers and the context surrounding aggressive disagreements; degree to which the child(ren) were exposed; and perceived consequences for adults and children.

**Results:**

All couples listed household responsibilities and parenting as key IPV triggers. Couples with infants reported that parenting disagreements were particularly heated, with women using aggression due to frustration about their partners’ lack of support. Couples also described substance use, wanting to be heard, and prior violence histories as triggers or as the background context for IPV episodes. Children were present during IPV and often intervened in conflicts involving severe violence. Parents’ perceptions of the effects of IPV on their children ranged from minimal to major emotional distress, with men describing more significant impact than women.

**Conclusions:**

When describing acute triggers, parents most commonly mentioned that arguments were instigated by concerns about the division of household labor and parenting, a finding that may have significant implications for intervention development; this was particularly notable for parents of infants. Our findings emphasize the need for innovative programs that help parents cope with the stresses of raising a family as well as programs that directly address the consequences of IPV for children.

**Electronic supplementary material:**

The online version of this article (doi:10.1186/1471-2458-14-1324) contains supplementary material, which is available to authorized users.

## Background

Intimate partner violence (IPV) is a prevalent public health problem that affects millions of families. IPV increases the risk of myriad health conditions; for example, IPV-related illness and injury costs the health care system in the United States over $4.1 billion per year [1].

Much of what is known about IPV comes from *quantitative* studies that often "count" acts of IPV without exploring in depth the circumstances surrounding the violence, thereby leaving critical questions unanswered. For example, large quantitative surveys have found that women perpetrate at least as many acts of IPV as men [2, 3]. This finding has caused significant controversy, as the quantitative studies that report equal rates of aggression fail to provide information about the motivations or feelings associated with violent acts. Thus, some researchers have suggested that the high rate of female-perpetrated violence is due to the situational context of IPV in which women are afraid of their partners and primarily use aggression in self-defense, whereas men generally are not intimidated by women’s use of IPV and use it themselves to control their partners [4–6].

*Qualitative* IPV studies initially focused solely on women’s perspectives [7]; this focus likely related to researchers’ desire to bring attention to women’s experiences with battering and the serious damage resulting from victimization, and to raise awareness about the magnitude and severity of the problem [7]. One researcher summarized that these early qualitative studies about women’s experiences with IPV demonstrated that "cultural constructions of romance and ‘perfect love’ serve the function of binding women in abusive relationships….[and that women] co-opted traditional gender narrative[s] by making excuses for their partners’ violence and internalizing expectations that they should nurture their romantic partner" (p. 184) [7]. Although more recent studies have explored men’s narratives about IPV events, few studies have examined the perceptions of *both* partners within a relationship. This omission hinders in-depth understanding of the greater context within which violent disagreements occur. Specifically, our literature search identified only a small number of studies which we feel are important to describe in detail to contextualize the current study [7–9], that used data from both partners to investigate the immediate triggers for violent episodes.

Boonzaier (2008) recruited from social service agencies in South Africa, interviewing 15 heterosexual couples who were involved in a relationship with IPV. Using a narrative approach informed by a feminist perspective, she described myriad underlying themes related to perceptions of self in the relationship, cycles of violence and use of power tactics. For example, she reported that the men often resisted the label of perpetrator and defended themselves as good people who had been wounded by their partners, whereas women portrayed themselves as victims and described loving their partners and hoping that the abuse would cease [7]. Boonzaier concluded that couples often drew on gender norms to describe and contextualize their relationships, and that participants sought to construct some sense of order in discussions about their volatile relationships [7].

Nemeth (2012) analyzed recorded telephone conversations between men incarcerated for IPV and their romantic partners, and examined descriptions of triggers of violent episodes [8]. Accusations about infidelity were the most common trigger for acutely violent interactions; these violent episodes often occurred when one or both partners were under the influence of alcohol or illicit drugs [8]. Given that the sample was incarcerated men, however, their acute triggers may differ from those occurring in a community based sample. In addition, neither Boonzaier nor Nemeth specifically recruited parents or investigated how both partners construct narratives related to their children’s exposure to IPV, which may be an important omission [10].

Rates of IPV are disproportionately high in families with children, and IPV has well-documented adverse effects on child health [10, 11]. Although not well elucidated, the higher risk for IPV in families with children may be related to the negative impact of child rearing on couple harmony due to greater demands associated with parenting responsibilities and financial stresses. Parenting also may force couples to examine their gendered expectations about household responsibilities, including establishing which partner takes primary responsibility for child care [12]. Effective interventions for families experiencing IPV are urgently needed, yet few programs have documented long term positive impact [13]. The small number of effective interventions may be due in part to the lack of a deep empirical understanding about the complex dynamics involved in violent relationships amongst parents [14].

Therefore, using semi-structured interviews with couples who were parents and who reported recent IPV, the overarching objective of this study was to explore deeply what happens when these couples disagree, and particularly what happens when they used aggression.

## Methods

### Sample recruitment

Fourteen individuals from seven male-female couples who had sought couple therapy participated in semi-structured interviews (Table 1). Specifically, the study sample was comprised of 23 to 49 year old males and females aged 26 to 46. Data about race/ethnicity were abstracted from the clinic’s intake, with some participants not reporting this information. Of those with race/ethnicity data, one man and one woman were Black, three men and three women were White, and one man and one woman was Latino. Four of the seven couples were cohabiting, two were married, and one was recently separated. In total, the couples had 16 children, with a mean age of 9.7 years (standard deviation (SD) = 5.8 years; range = 4 months to 18 years). The modal of number of children per family was 2.29 (range 1 - 5). Four couples had younger children (infancy to grade school), and three had adolescent children. Participants were recruited from a family therapy clinic housed in a University located in a diverse community, with the therapists helping the research staff to identify potentially eligible participants. As per clinic policy, if during the intake assessment either member of a couple reported IPV resulting in physical injury requiring medical treatment or expressed fear of living with the partner or participating in therapy, couple therapy was not provided, but the partners were offered individual therapies and given appropriate IPV referrals; therefore, couples with this type of violence were not eligible for interviews.

**Table 1:** **Characteristics of couples and their children**

Couple ID	Female partner		Male partner		Cohabitation status	Marital status	Number of children	Sex and age of children
	***Age***	***Highest education***	***Age***	***Highest education***				
1	34	11^th^ grade	Not given	Some college	Living together	Not married	1	Female, 9 years
2	40	12^th^ grade	47	Some college	Living together	Not married	2	Female, 16 years
								Female, 16 years
3	26	10^th^ grade	24	GED	Living together	Not married	1	Male, 2 years
4	26	Some college	23	12^th^ grade	Living together	Not married	2	Female, 2 years
								Male, 4 months
5	47	PhD student at time of interview	49	12^th^ grade	Living together	Married	4	Female, 18 years
								Male, 16 years
								Male, 14 years
								Male, 10 years
6	28	College student at time of interview	31	Technical school graduate	Separated	Married	1	Female, 4 years
7	36	High School	41	9^th^ grade	Living together	Married	5	Female, 15 years
								Female, 12 years
								Male, 9 years
								Female, 7 years
								Female, 4 years

Given the sensitive content of the interviews, our sampling strategy represented a combination of convenience and purposive sampling. Convenience sampling strategies allow researchers to recruit a group of participants to whom they have easy access; in our case, recruiting from a therapy program was both the most accessible and safest approach. However, consistent with qualitative methods, we also employed purposive sampling which has been defined as "identifying and selecting individuals or groups of individuals that are especially knowledgeable about or experienced with a phenomenon of interest" [15]. Specifically, we employed criterion sampling, a sub-type of purposive sampling that involves choosing cases that meet pre-decided criteria thereby providing an in-depth understanding in the subject of interest [15]. For our study, the criteria included that couples had to: 1) be heterosexual and English-speaking; 2) both at least 18 years old; 3) have experienced severe verbal aggression or any physical aggression within their relationship (enacted by one or both partners) during the past 4 months, as assessed by reports to their therapist, or by their written self-report of partner aggression on measures described below; and 4) have one or more children up to age 18 years of age living in the household.

### Measures

As a part of the clinic’s pre-treatment assessment, the members of a couple independently complete questionnaires. The following measures from this assessment were used *solely to identify* couples who met IPV inclusion criteria:

*Revised Conflict Tactics Scale* (*CTS2* [16]): The CTS2 assesses IPV acts that have occurred during the past four months and contains sub-scales that assess a range of severity of psychological aggression and mild to severe physical violence. Couples were eligible if either member reported perpetrating or receiving any severe verbal or any physical aggression. Alpha reliability coefficients for the male-to-female aggression scales range from 0.79-0.91 and female-to-male 0.82 [16].

*Multidimensional Measure of Emotional Abuse (MMEA)* [17]: The MMEA has four subscales assessing forms of psychological aggression: denigration, domination/intimidation, hostile withdrawal, and restrictive engulfment. The MMEA subscales have Cronbach alphas ranging from .80-0.92 [18] Couples were included if they disclosed any incidents of psychological aggression during the past four months.

### Procedure

When a couple was identified by their therapists as meeting inclusion criteria, the therapist informed each person individually about the study and asked if they were interested in being contacted by a research assistant (RA). If both were interested, the RA arranged a mutually agreeable time to meet at the family therapy center. Each participant provided written informed consent that explained that the purpose of the study was to understand what happens when things do not go well when couples are fighting, and particularly what happens when couples use aggression. This study was approved by the following Institutional Review Boards: Johns Hopkins School of Medicine, University of Maryland-College Park and Boston Medical Center.

Each person participated in interviews separately for safety purposes and to minimize the potential for each person to influence the other’s responses. Interviews were semi-structured, using an interview guide created a priori based on our review of the relevant literature regarding processes commonly occurring in IPV (Additional file 1). Questioning was also dynamic, allowing for the expansion of emerging themes. Key topics included: 1) types of aggression used; 2) primary triggers and background context surrounding aggressive disagreements; 3) degree to which the couple’s child [ren] were exposed to the violent episode; and 4) perceived adult and child consequences of the aggression. Interviews lasted approximately one hour. Each participant was remunerated with a $50 gift card.

All interviews were audio-recorded to facilitate verbatim transcription and data analysis. Recordings were transcribed by a professional transcription company and were checked for accuracy (by author MM). All coding was conducted using Atlas.ti software (Atlas Corporation, Berlin), which allows researchers to systematically organize and qualitative data for key content and organize complex relationships.

Consistent with the steps of thematic analysis, transcripts were coded in a systematic, iterative fashion based upon the framework proposed by Miles and Huberman (1994) [18]. The first step in this framework is *data reduction*. An initial set of a priori codes were generated based on the interview guide. However, additional codes were added as the authors read and listened to the transcripts. Three authors, all of whom had read and studied the text, met on multiple occasions to discuss and refine the code list, examine code output, and worked collaboratively to define and document salient themes that emerged from the iterative coding process. These authors then went back to the interview texts to review and confirm their interpretation of the main themes identified.

For the second step - *data display* – data were organized through the use of charts that permitted comparing and contrasting code output, salient themes and discerning patterns [18]. We explored similarities and differences in themes by sex and within dyads. For example, charts were created to compare acts of reported violence by sex. During the final step – *conclusion drawing and verification*–, we considered the meaning of the results, with the data revisited to confirm conclusions [18].

## Results

### Types of violence

Within dyads, men and women concurred that psychologically and physically violent acts were largely initiated by women, with men responding with reciprocal – and for some couples more severe - violence. The reported violent acts spanned the gamut from insults to destroying property to choking, with three patterns of violence emerging between dyads. Three couples (couples 1, 3 and 4) primarily used physical violence (e.g., hitting with fists, pushing, and threatening with a knife) in their disagreements. The other four dyads (couples 2, 5, 6 and 7) described psychologically aggressive arguments that occasionally escalated to either property damage or to physical aggression (e.g., choking, pushing, or throwing hot liquids at one’s partner). For example, couple 7 described the woman yelling at her partner about a household chore, and then taking her wedding ring off and throwing it at him. In response, he took one of her "collectible dragons," dropped it on the floor and stomped on it.

### Aggressive conflicts: triggers and context

In narrating their violent altercations, participants both described the disagreement that immediately preceded the violence and explored the broader relational and situational context within which violence occurs within their relationships. The following sections describe the core triggers and contexts.

#### Parenting/household responsibilities

Most of the narratives focused on dissatisfaction with the division of parenting and household labor, with both men and women attributing the acute trigger of violent acts to their perceptions of lack of support or unequal division of household and parenting responsibilities. Whereas women with older children focused mostly on the conflicts associated with the expectation that they alone should handle a wide array of household tasks, women with younger children portrayed their lives as imbued with adversity (e.g., post-partum depression; substance use), explaining that within this broad background of major stressors their partners’ failure to take enough responsibility in child rearing or within the household was the proverbial "last straw." The peri-partum period appeared to be a time of high risk for some mothers, who described intense post-partum rage leading to aggression. One woman (couple 3) described, *"I was going through postpartum very bad. It was late, and I was wondering-- it’s like midnight. I’m home with our son, our newborn. Where the hell are you? …I go out riding around trying to find him. I find him at [a bar]..I’m like, ‘We got a newborn at home; you should be home with me, and not running the street,’ and then he comes home, and we just get into it… I’m still going off…and, next thing I know he’s choking me, and I got the baby in my arms and he spit in my face."* This woman’s partner recapped the same incident as follows: "*I get a call from her saying that she’s upset because he’d [the baby] been crying… and she expected me to come home, and I wasn’t ready to come home…She yelled at me and was cursing…then I hear the window get smashed upstairs because she had taken a chair and thrown it at the window…. She went off on this wild tangent of saying what a bad dad I am and how lazy I am….and I snapped. I grabbed her by the throat. I choked her. I was insulted."*

Men’s statements regarding parenting and the division of labor were largely about their partners’ lack of paid work. They contrasted this with their own sense of taking financial responsibility, touting their role as "bread winners" and expressing their frustration at their partners’ lack of appreciation of this role or lack of financial contribution to the household. The man from couple 3 stated, *"She’d do whatever she wanted pretty much, and I worked and did what I could to try to support and pay for her fun and what not, and I guess in some ways I just kind of held resentment to that because– especially when (our son) came into the picture because it just never really seemed like she was really trying to really step up….I had gotten a GED. I got a better job. I was trying to do what I could to improve my footing in life… sometimes I’d call her from work, and she’s taking a nap, and then of course I’m – come home, and we’d fight."*

Couples also described differing notions of being a "good" parent as a source of underlying tension that fueled more general feelings of anger with their partners. Couples disagreed about how to best interact with their children and about how to set boundaries for their children’s behaviors; this parenting-focused anger served as a backdrop for later aggressive conflicts. The man from couple 3 continued, *"…it seemed like she was being kind of lazy with him (our son), like she would just sit in front of the computer on Facebook or whatever and just ignore him and put him in front of the TV, let him – let that babysit him, and if he got fussy in the slightest little bit, she would yell at him and be mean to him, and I don’t like that because he’s just a little boy."* A woman from another dyad (couple 5) stated, "*We should be on the same page [about parenting] and if I told them that they can’t do this, you can’t tell them they could do something that I told them not to do…And I’m fussing when I come in the door. I work all day and I come home and you [the children] all got food laying here….And my husband says ‘Don’t come in here fussing. Leave them kids alone.’ I’m like, well, do you want to let them raise themselves. So we argue about that. He started yelling first…. He’ll say things like, you’re so mean, you’re so aggressive*." The woman then stated that she proceeded to purposefully say things to her husband that she knew would be hurtful to him.

#### Substance use

Aggressive altercations emerged within a greater environmental milieu of chronic stressors and risk-taking behaviors. Many couples described aggression occurring predominantly while under the influence of alcohol or drugs, while others specified that anger related to their partner’s substance use (or actions while under the influence) contributed directly to the aggressive conflict. One man (couple 1) said that their most physically violent disagreement occurred when his partner "*was so high on pills that she didn’t know what was going on…"* Another man (couple 4) stated, "*I got drunk, you know what I mean, I passed out…. I could barely move, but I was still conscious, but I could tell, she started getting madder, started smacking me around, trying to get up. I told her to chill. Then she came, she started throwing water at me. So I was like, Yo, relax…Then she went and she pulled out a knife on me."*

#### Wanting to be heard

Women often described loving their partners but feeling extremely frustrated at their partners’ lack of attention. They expressed the need to use physical violence or to damage property to elicit a reaction from their partners. One woman stated that, although she does not consider herself a violent person, she threatened her partner with a knife during three separate arguments so he would stop yelling and listen to her. Another woman (couple 6) described, "*What (destroying his things) does for me is…I’m trying to get a reaction out of him. I want him to feel the amount of hurt or disappointment that I feel, and the only way…I can get that…is by damaging the things he cares about*."

#### Prior experiences with abuse

Respondents struggled to make sense of their aggressive disagreements, and often contextualized them within the background context of prior experiences with abuse. Although the interview questions did not ask about prior exposure to abuse, multiple participants spontaneously discussed prior personal (e.g., sexual or prior intimate partner) abuse or exposure to IPV in their families of origin. For example, one woman from couple 1 described an altercation, triggered by a parenting disagreement, in which her partner broke her nose. Describing the conflict, the woman spontaneously interjected the following about daughter about which the parenting disagreement was focused: *"A’s like my heart because her dad [a former partner] and I, when we were together he was very, very abusive to me and I ended up miscarrying her twin when he threw me down…he traumatized me very much to where it makes it hard for me to try to enjoy any kind of relationship at all…"*

### Child involvement and consequences

When participants discussed their violent disagreements, they described that their children often were present and were involved in myriad capacities ranging from attempts to distract their parents to calling to the police. Children were more likely to be directly involved when significant physical violence was occurring; specifically, the children of the three couples who primarily used physical aggression all stated that their children had tried to intervene to stop the disagreement, with one child calling the police, and others trying to physically separate or otherwise distract their parents. One woman (couple 6) said, *"… sometimes it’s like if she senses that I’m angry or he’s angry, she’s so smart. She’ll try and intervene. Or she’ll start talking about something and get louder so that our attention comes back to her…She’s like, ‘Mommy, my foot is itching me’, or just anything, just to get the attention off of what may occur."*

Parents’ perceptions of the effects on their children of witnessing IPV ranged from minimal to major emotional distress. When parents remembered more intensely physically violent disagreements, however, they also were more likely to report more intense negative child reactions. In their attempts to understand the meanings of IPV exposure on their children, the men involved in the most violent relationships were most likely to describe concern that aggression had negative effects on their children. For example, the father from couple 1 described the following child response: ***Interviewer (I)****: When this fight was going on, where was E [9 year old daughter]?****Father (F)****: She was there, and she was terrified.****I****: What was she doing?****F****: She’s the one that called the police. She was visibly upset*. A second father (couple 3) explained: "*He [2 year old] kind of does his little wail, and he kind of runs up to us….. I think he’s upset that we’re fighting and yelling at each other and he wants us to stop, and then he wants to make sure that neither of us get hurt.*" In contrast, women in general were more likely to report that either their children were not aware of the disagreements or that the disagreements were not particularly upsetting.

### Consequences for adults

Women expressed feeling emotionally hurt after incidents of violence. Most interpreted their partners’ words and actions from these disagreements as evidence of their inadequacy, decreasing their perceived self-efficacy and in turn increasing their feelings of desperation to be heard. The woman from couple 7 shared "*I can’t stand fighting with him. And some of the things they say they just make you feel belittled. And it’s like, ‘Really’? Downgrade you. And it’s just a simple little word and it just makes you feel like ‘Wow you really think of me like that. Great’. … That stabs you right in the heart.*" Some women also described the intense fear that they felt while these disagreements were occurring. The woman from couple 3 reported, *"When he choked me that’s where I thought he really messed my neck up. I mean he– I thought I was going to die. I really did. I thought I was going to die."*

The men generally did not report fear of their partners, but rather appeared surprised that their partners would employ violent tactics. When asked his feelings after his partner pulled a knife on him, the man from couple 3 responded, "*I was just stunned. It was just like "Oh, my god, I can’t believe you are pulling a knife on me…"* None of the men reported feeling saddened as a result of violent disagreements, but several expressed concern that chronic stress resulting from violent disagreements would manifest as somatic symptoms. For example, when discussing the prospect of staying in the relationship for the sake of his daughter, the man from couple 1 stated "*Because you might be trying to do the right thing, but in the long run…it’s a 50/50 shot. And why risk your happiness and maybe having a heart attack or something like that because you’re so stressed out, for something that’s probably not going to work?"* When asked directly how violent disagreements affected his health, another man (couple 5) replied *"My health? Well, I mean, I have been in fights before, but it’s stress. It’s just stress, just a lot of stress, and that leads to all kinds of illness…*"

## Discussion

This qualitative study analyzed narratives from a sample of parents who were seeking therapy, focusing on understanding what happens when couples disagree, and particularly what happens when they use aggression. A number of our findings illustrate the complexity of such relationships and merit further exploration. When describing acute triggers, parents most commonly mentioned that arguments were instigated by concerns about the division of household labor and parenting, a finding that may have significant implications for intervention development. Couples with infants reported that parenting disagreements were particularly heated, with women using aggression due to frustration about their partners’ lack of support. Parents also described additional stressors (like personal histories of violence or substance abuse) that served as important context for the acute episodes of violence. Children were present during aggressive disagreements and often intervened in conflicts involving severe violence. Parents' perceptions of the effects of aggression on their children ranged from minimal to major emotional distress, with men describing more significant impact than women.

In prior studies, marital satisfaction has been inversely correlated with number of children and with discord between spouses with regard to distribution of household tasks [12, 19]. Our findings suggest that programs seeking to prevent or mitigate IPV amongst parents must specifically address parents’ perceptions about the division of household labor, as well as the additional responsibilities brought on by having children. Particular focus on this area may be of particular importance for couples reporting cumulative background stressors.

Myriad studies have described the considerable prevalence of postpartum depression or blues, and have investigated the socio-demographic factors that increase women’s risk [20, 21]. Some women in the current study referred to their "postpartum" time as a period of extreme stress and as a period in which they needed more support from their partner but did not receive it [20, 22]. These women talked about the unrelenting work and fatigue associated with a newborn, and resented the imbalance of household responsibilities. Although some studies refer to lack of social support as a risk factor for postpartum depression or blues, we are unaware of studies that describe lack of partner support as a direct cause of women’s IPV perpetration during the postpartum period. This finding speaks to the tremendous need for the expansion of interventions worldwide, like early childhood home visitation [23], that support women during the peri-partum time period. Early childhood home visitation has become a priority in the United States, and is supported under the Patient Protection and Affordable Care Act of 2010. Although the evidence-based early childhood home visitation programs vary to some degree in content and in mode of delivery, all seek to optimize maternal and child health around the time of a new birth [24]; state-based programs receiving federal funds are required to show positive impact in key areas, including reduction of IPV.

Despite the fact that both men and women reported female-initiated violence, the consequences of that violence differed in important ways between sexes. Even though women reported using what has classically been categorized as more severe violent tactics, men did not report feeling either fearful or saddened. Women, on the other hand, expressed feeling afraid for their well-being when men were violent toward them. In addition, women described connections among their partners’ violent acts, their associated fear, and their general feelings of sadness. Women’s fear is likely due in part to male-female size and strength differences; even studies that have found that women use as much violence as men generally have indicated that men are more likely than women to use violence that leads to injury [2]. These results mirror existing quantitative literature examining how IPV differentially affects men versus women. For example, using data from a Canadian telephone survey, Ansara and Hindin found that women were more likely than men to experience severe violence at the hands of their partners, and that men more commonly reported that the IPV did not adversely impact their health [25]. Similarly, Kamimura and colleagues found that women, and not men, who had experienced IPV reported worse psychological functioning [26]. Thus, our results add support to the general finding that, regardless of the types of violent acts that are used, the impact of violence on well-being may be different for men and women.

Parents reported that their children appeared distressed by and/or were directly involved in their aggressive disagreements. This finding is concordant with Davies’ and Cummings’ emotional security theory [27–29] which states that children take cues from the inter-parental relationship about whether their parents will be able to preserve the integrity of their family and ensure the child’s safety; as the couple’s conflict becomes more aggressive, children’s concern for safety increases, leading them to experience negative emotional reactivity and to engage in attempts to regulate exposure (e.g., through directly intervening) [29]. While our interviews document parental perceptions of the impact of aggressive disagreements on children, empirical literature provides objective support that increased involvement places children at risk for traumatic reactions and for being physically injured. It is noteworthy that, in the couples disclosing the most intense physical violence, the fathers, and not the mothers, reported concerns about their child’s emotional reaction. This may be based in part on societal pressure for women to be considered "good mothers." In general, in US society, mothers are more likely to both take responsibility, and be held accountable, for their children’s well-being. Mothers may have been concerned that admitting to themselves or to a researcher that their violent altercations had upset their children may be perceived as a sign of inadequacy, and lead to blame focused on them [30]. A second possible explanation is that the participating men were aware of the gender stereotypes that cast men as the primary aggressors, and felt the need to refute this stereotype by portraying themselves as sensitive to the impact of IPV.

The results of the present study should be interpreted within the context of several limitations. First, qualitative studies are not designed to be generalizable but rather are intended to improve understanding of specific phenomena within a circumscribed sample. Our sample was drawn from a clinic serving a diverse population, but the sample size was relatively small, and the couples had been screened to exclude those who had experienced IPV resulting in physical injury; in addition, we used a sampling approach that united both convenience and purposive sampling, which may have limited our ability to capture potential participants with information-rich stories to share. Second, respondents may have provided socially desirable responses, given that the topic involved violence. However, couples did readily disclose significant acts of aggression as well as illegal behaviors.

## Conclusions

The frequency with which couples cited an imbalance of parenting/household labor as a major trigger for violence warrants further exploration and provides insight for intervention targets. Increasingly, innovative programs like early childhood home visitation are providing support for parents. Additional examples of evidence supported parenting programs can be found on the Child Welfare Information Gateway ( https://www.childwelfare.gov/topics/preventing/programs/). This study supports the tremendous need for developing IPV prevention programs and interventions that help families balance the multiple demands of work, household functioning, and parenting, particularly when they are facing other background stressors.

## Electronic supplementary material

Additional file 1: **Interview Guide.** (DOC 30 KB)

## Abbreviations

IPV: Intimate partner violence
CTS2: Revised Conflict Tactics Scale
MMEA: Multidimensional Measure of Emotional Abuse
RA: Research assistant.
