# Supplementary material for: The context of violent disagreements between parents: a qualitative analysis from parents’ reports
Source: BMC Public Health. 2014 Dec 24;14:1324. doi: 10.1186/1471-2458-14-1324 (PMC4506431; doi:10.1186/1471-2458-14-1324)
Supplement: Supplementary file 1 — Additional file 1: Interview Guide. (DOC 30 KB) [file 12889_2014_7442_MOESM1_ESM.doc]

**Additional file 1**. Interview Guide

**Semi-structured Interview Guide**

Thank you for taking the time to talk to me. Before we begin our chat, I’d like to ask you a few basic questions about yourself and your family.

1. Mark gender of participant: __________Male ________________Female
2. How old are you?
3. What is the highest grade of school that you have finished (#/University)?
4. Do you work outside of the home? What do you do?
5. Are you currently living with your partner?
6. Are the two of you married?
7. Do you and your partner have children together?
8. How many children do you have in total? __________ (#)
9. How old are they? ___ (#); ____(#); ____(#); ____(#);____(#)
10. How many people live with you in your home right how? _____________ (#)
11. Tell me who are the people that you currently live with? (Describe relationship to participant e.g. partner/child/others)

Now I’d like to hear a bit about what is going on for you and your family right now. Please feel free to be as open as possible.

Let’s start by talking a bit about some of the challenges you and your family might be facing. Those may include the concerns that led you to contact the Center for Healthy Families for assistance, but they can include other challenges as well.

Please tell me about some of the problems that you and your family are dealing with.

*Probes/ prompts*: What types of things have been weighing on your mind these days?

I understand that you and your partner are having some challenges related to resolving arguments. Please tell me a little bit about that….,.,

What do you generally argue about?

How do these arguments generally start?

What happens when you argue and things do not go so well?

What do you remember happening during the last time things got so heated that one of you hit, punched, kicked, pushed or otherwise used aggressive behavior toward the other. Tell me what happened…who did what first, and what happened next, and so on….

When did this last happen? _____________________ (# days/weeks/months ago)

Where were you when this happened?

In what ways were you and your partner physically aggressive?

*Probes/ prompts*: Who [pushed, grabbed, hit, kicked, punched, etc] who first? Did [you/partner] also [hit, punch, etc]?

Were there things prior to this that led up to this fight? What was going on before this fight? The week before? The day before?

Tell me about what happened just before [you/your partner] [pushed, grabbed, hit, kicked, punched, etc].

Why do you think you [pushed, grabbed, hit, kicked, punched] your partner?

Why do you think he/she [hit, kicked, punched] you?

*Probes/ prompts*: How would you finish this sentence: I (he/she) [hit, kicked, punched] me/him/her because……..

What were you thinking and feeling when the fight was going on? What was going through your head? What do you think was going through your partner’s head?

How did you feel about your partner [hit, kick, punching] you? How do you think he/she felt about you [hit, kick, punching] him/her?

How have drugs or alcohol influenced your relationship with your partner and your ability to get along?

*Probes*: Have drugs or alcohol ever played a role in those times where your fights have become physical? Tell me more about that…

Did anyone get hurt?

*Probes/ prompts*: Who? In what way did they get hurt?

How did the fight end?

How did you feel at the end?

How do you think this fight or others like it is affecting your health?

How would you rate this fight compared to other times in the past several months when you and your partner have disagreed or argued?

*Probes/ prompts*: How was it more or less intense?

Who else was around that might have heard or seen the fight?

*Probes/ prompts*: Where were the children?

[If kids present] How did the kids react? Did they get involved in some way?

[If kids not present] Do you think that they picked up later that something had happened? Why or why not?

How do these heated arguments where one or both of you [hit, kick,push] the other one affect your children?

*Probes/ prompts*: ASK SEPARATELY ABOUT EACH CHILD.

How do you think ___________(name) feels about the fighting between you and your partner (name each)? In what ways do you see (name each) being affected by this type of fighting?

Tell me a bit more about why you think things are different for (child A) vs (child B)?

In general, how do you think that a child would feel if he or she saw her Dad hitting her Mom? How about his/her Mom hitting her Dad? Would these two be different for the child?

Tell me about any other concerns you might have for your child(ren) as it relates to your relationship with your partner?

We have spent some time talking about what happens when things do not go well with your partner. I was hoping that you could tell me a little bit about how you resolve disagreements so that things are able to be resolved without yelling or fighting?

What types of things do you think help you and your partner to resolve your disagreements so that things don’t get heated? What seems to work best between the two of you? What positive approaches would you like to see the two of you use more?

We are coming to the end of our time together today. I really want to thank you for talking with me and sharing your experiences. Is there anything else you would like to share at this time?
